# *RFC1* AAGGG repeat expansion masquerading as Chronic Idiopathic Axonal Polyneuropathy

**DOI:** 10.1007/s00415-021-10552-3

**Published:** 2021-04-21

**Authors:** Matteo Tagliapietra, Davide Cardellini, Moreno Ferrarini, Silvia Testi, Sergio Ferrari, Salvatore Monaco, Tiziana Cavallaro, Gian Maria Fabrizi

**Affiliations:** Department of Neurosciences, Biomedicine, and Movement Sciences, University of Verona, Policlinico G.B. Rossi, Piazzale L.A. Scuro 10, 37134 Verona, VR Italy

**Keywords:** Chronic Idiopathic Axonal Neuropathy, Replication factor C subunit 1, Cerebellar Ataxia, Neuropathy, Vestibular Areflexia Syndrome

## Abstract

**Background:**

A biallelic intronic AAGGG repeat expansion in the Replication Factor C subunit 1 (*RFC1*) gene has been recently associated with Cerebellar Ataxia, Neuropathy, Vestibular Areflexia Syndrome, a disorder often presenting as a slowly evolving sensory neuropathy at the onset. “Chronic Idiopathic Axonal Polyneuropathy” (CIAP) is a common indolent axonal neuropathy of adulthood which remains without an identifiable cause despite thorough investigations.

**Methods:**

We screened 234 probands diagnosed with CIAP for a pathogenic biallelic *RFC1* AAGGG repeat expansion. Patients were selected from 594 consecutive patients with neuropathy referred to our tertiary-care center for a sural nerve biopsy over 10 years.

**Results:**

The *RFC1* AAGGG repeat expansion was common in patients with pure sensory neuropathy (21/40, 53%) and less frequent in cases with predominantly sensory (10/56, 18%, *P* < 0.001) or sensorimotor (3/138, 2%, *P* < 0.001) neuropathy. The mutation was associated with sensory ataxia (*τ*_b_ = 0.254, *P* < 0.001), autonomic disturbances (35% vs 8%, Prevalence Odds Ratio—POR 6.73 CI 95% 2.79–16.2, *P* < 0.001), retained deep tendon reflexes (score 18.0/24 vs 11.5/24, *R* = 0.275, *P* < 0.001). On pathology, we observed absent/scant regenerative changes (*τ*_b_ = − 0.362, *P* < 0.001), concomitant involvement of large (100% and 99%, n.s.), small myelinated (97% vs 81%, POR 7.74 CI 95% 1.03–58.4, *P* = 0.02) and unmyelinated nerve fibers (85% vs 41%, POR 8.52 CI 95% 3.17–22.9, *P* < 0.001). Cerebellar or vestibular involvement was similarly rare in the two groups.

**Conclusions:**

This study highlights the frequent occurrence of the *RFC1* AAGGG repeat expansion in patients diagnosed with CIAP and characterizes the clinical and pathological features of the related neuro(no)pathy.

**Supplementary Information:**

The online version contains supplementary material available at 10.1007/s00415-021-10552-3.

## Introduction

Chronic polyneuropathy is a common neurological disorder, occurring in at least 4% of the middle-aged and elderly population and increasing in prevalence with age. Current estimates report a missing etiology in as many as 11–45% of patients with chronic polyneuropathy, especially in axonal forms [1, 2]. Chronic Idiopathic Axonal Polyneuropathy (CIAP) is formally identified in the presence of (a) clinical signs of distal sensory or sensorimotor neuropathy with little progression over a 6-month time window, (b) large fiber axonal neuropathy as observed on nerve conduction studies (NCS) and (c) absence of an identifiable underlying etiology. Although a lack of a clear inheritance pattern is currently assumed for a CIAP diagnosis [3], recent reports indicated that ‘idiopathic’ late-onset axonal neuropathies do have a mendelian inheritance in some cases [4].

Cerebellar Ataxia, Neuropathy, Vestibular Areflexia Syndrome (CANVAS) is a complex and presumably rare ataxic disorder occurring in middle-aged or older individuals, recently associated to a biallelic intronic pentanucleotide repeat expansion of a mutated AluSx3 element (AAGGG^exp^) of the Replication Factor C subunit 1 (*RFC1*) gene (Online Resource 1) [5]. First identified by neurotologists as a combined degenerative disorder of cerebellum and vestibulum [6], the disease was later renamed to encompass the frequent peripheral sensory disorder [7], an ubiquitous feature of the full presentation as shown in the largest retrospective CANVAS case series available to date [8].

Here we report a retrospective cross-sectional study examining the prevalence of the *RFC1* expansion in biopsy-investigated CIAP patients according to their clinical presentations. As a secondary analysis, we determined the differences of clinical features, instrumental investigations and sural-nerve biopsy findings between *RFC1* cases and non-mutated patients.

## Materials and methods

### Recruitment criteria

Two readers (M.T. and D.C.) reviewed the pathology register at Neuropathology laboratory, Azienda Ospedaliera Universitaria Integrata, Verona, for patients who underwent sural nerve biopsy between 01.01.2007 and 31.12.2016 and were older than 18 years at the time of biopsy. In accordance with clinical practice, thorough investigations for sensory and sensorimotor neuropathies are required before referral (nerve conduction studies and blood panel for rheumatologic, immune, infectious, metabolic and hematologic causes of neuropathy) [9]. Two neurologists (T.C and S.F.) evaluated every patient at the time of sural nerve biopsy.

To only include patients receiving a final diagnosis of CIAP, historical records were scrutinized to exclude patients with, in hierarchical order, (a) a definite alternative diagnosis; (b) acute onset; presence on pathology studies of (c) inflammatory or (d) demyelinating changes; (e) focal alterations on clinical examination and/or pathology; (f) absence of relevant alterations on pathology. We further excluded CIAP patients without an available sample for genetic analysis. Biopsy of multiple individuals from the same family are a very rare occurrence in our practice and were not observed in this particular sample.

### Data collection

Each reader independently abstracted the following data from the clinical records: sex, age at onset, apparent sporadic or familial occurrence, disease duration, age at biopsy and patterns of clinical involvement. “Sensorimotor” cases were defined by a Medical Research Council (MRC) score ≤ 3/5 in at least one muscle group bilaterally, not otherwise explained; “predominantly sensory” cases had evident motor involvement not fulfilling the previous definition; “pure sensory” cases had no evident motor involvement. Sensory impairment was graded according to Sensory Modality Sum Score (SMSS) [10, 11] (pinprick sensation was amended as it is not systematically assessed at our Clinic). Ataxia was evaluated according to an in-house impairment scale (0 = normal, 1 = mild ataxic signs, 2 = overt ataxic gait, 3 = uses unilateral walking aid, 4 = uses bilateral walking aids/wheelchair). Ankle jerks, patellar, brachioradialis and biceps deep tendon reflexes (DTR) were graded as follows: 0 = normal, 1 = just elicitable, 2 = dull, 3 = normal, 4 = brisk, 5 = frank hyperreflexia or clonus. The presence of paresthesias/tingling in any site, Romberg sign, cerebellar signs, nystagmus, vestibulopathy, dysphagia, cough, restless legs syndrome, pes cavus, hammer-toes, neuropathic pain, autonomic disturbances were also investigated, either reported as suggestive symptoms or detected on dedicated testing.

Last NCS available prior to biopsy was assessed for the pattern of involvement (axonal/demyelinating/mixed) and for sural sensory nerve action potential (SNAP) amplitude of the biopsied nerve. If available, CSF results were collected.

All the biopsy specimens had been previously analyzed during routine neuropathological practice by the same trained neuropathologist (T.C.). Historical reports were abstracted for the involvement of large and small myelinated fibers (MF) (apparent reduction in fiber density on a qualitative analysis of toluidine blue semithin sections observed by light microscopy) and unmyelinated fibers (apparent reduction in fiber density, presence of collagen pockets and denervated Remak cells on a qualitative analysis of ultrathin sections assessed by transmission electron microscopy). Likert-scales were used to quantify degenerating fibers, regenerative changes (0–3 points) and microangiopathy (0–2 points). All aforementioned data extraction was done previous to genetic analysis, and discordance between the two readers was resolved by consensus.

### Genetic analysis

Repeat expansion analysis in candidate probands was performed by a single operator (M.F.), blinded to clinical data, as described in a previous study [5]. Genomic DNA from peripheral blood leukocytes was extracted by the standard salting out procedure. At first, all specimens were tested by flanking PCR to investigate the presence of a 348 base-pairs product corresponding to at least one not-expanded allele in the *RFC1* gene (Online Resource 2). Patients that failed to amplify were further analyzed by three distinct repeat-primed PCR (RP-PCR) for repeat expansions associated with the prototypical pathogenic (AAGGG) motif or with the (AAAAG) and (AAAGG) motifs (Online Resource 3). RP-PCR were performed using a GoTaq^R^ Long PCR Master Mix (Promega); fragment length analysis was done on a CEQ 8800 DNA analyzer with Fragments software (Beckman Coulter); a “sawtooth” pattern is observed only in the RP-PCR reaction specific for the (AAGGG) motif identifying patients with the biallelic AAGGG^exp^ associated with CANVAS, herein abbreviated ‘*RFC1*-positive’ (Online Resource 4). In *RFC1*-positive patients, a Next-Generation Sequencing (NGS) panel was used to rule out concomitant mutations in genes associated with hereditary neuropathies (Online Resource 5).

### Exploratory morphometric analysis

Sural nerve biopsies of *RFC1*-positive cases were subsequently reviewed. Semithin (1.0 µm thick), cross-sural nerve sections stained with toluidine blue were assessed on an optical set (ZEISS Axioscope 5 light microscope). Images were acquired using a coupled ZEISS Axiocam 208 color digital camera on a final optical magnification of 50 × for nerve fascicles and 400 × for nerve fibers evaluation (additional digital magnification set by the operator), and analyzed using ZEN 3.2 software by a single operator (M.T.).

Total cross fascicular area was defined as the area enclosed by the inner perineurial sheath. Axonal area of each fiber was defined as the area enclosed by the inner perimeter of the myelin sheath, excluding and counting separately regenerative clusters, degenerating fibers and non-measurable fibers. We analyzed at least three nerve fascicles from each sample to cover at least 30% of total endoneurial area and 100 measurable fibers, up to the whole nerve. To harmonize with previous literature, axon size is presented as the diameter required to describe a round axon of the same area.

The total number of MF was estimated by multiplying fiber density and total cross fascicular area. We opted to analyze the total number of MF instead of fiber density as the former variable is less influenced by healthy aging [12, 13].

On a Gaussian mixture assumption, an Expectation–Maximization iterative algorithm can be applied to obtain the characteristic parameters of the theoretical bimodal distribution of MF axon diameters in adult sural nerves. However, extremely low nerve fiber counts in our population prevented this direct approach. Instead, four control specimens from patients with acute mononeuritis multiplex deemed normal on pathology (a 48 and a 65-year-old male; a 50 and a 72-year-old female) were sampled with a multiple windows approach to obtain data on a pooled population of ~ 2000 fiber. The algorithm was applied to this population to compute the five descriptive parameters (two means *μ*_1–2_, two standard deviations *s*_1–2_ and the mixture proportion *λ*). Two axon size intervals (*μ*_1_ ± *s*_1_ and *μ*_2_ ± *s*_2_) were then identified and adopted to estimate the two subpopulations of small and large MF in pathologic cases, as a tradeoff between data consistency and minimal subpopulation overlap.

### Data analysis

We computed the following variables: proportion of total, small and large MF in *RFC1*-positive compared to reference values. Numerical variables were assessed with histograms, Q-Q plots and Kolmogorov–Smirnov’s test for normality. If appropriated, quadratic or logarithmic transformations were adopted.

First, patients were categorized according to clinical presentation. Group differences in demographical variables were checked using *χ*^2^ or Kruskal–Wallis one-way ANOVA non-parametric test followed by post hoc Mann–Whitney’s *U* as appropriated. Association between clinical phenotype and mutation status was assessed by Fisher’s exact test, followed by a post-hoc pairwise *z*-test for independent proportions; Bonferroni method was adopted to control for type I error inflation.

To explore clinical and instrumental elements suggestive for *RFC1* expansion mutation, patients were grouped according to the presence of biallelic AAGGG^exp^ and tested for differences (a) in frequencies on sex, clinical, instrumental and pathology variables with *z*-test for independent proportions and (b) in median age at onset, disease duration, SMSS, DTR score, semiquantitative pathology scales with Mann–Whitney’s *U* test, as normality could not be assumed. Effect size were described as (a) prevalence odds ratio (POR) for binomial variables; (b) Cramer’s V for categorial variables; (c) Kendall’s Tau b for ordinal variables; (d) Spearman’s Rho for continuous variables, as normality could not be assumed.

Relationships between morphometry and clinico-demographical variables were analyzed on scatterplots; linear relationships were then tested with Pearson’s correlation coefficient for normally distributed variables or alternatively Spearman’s rank correlation test. Difference between upper and lower limb involvement was tested with Wilcoxon signed-rank test. Significance was set at *α* < 0.05, each analysis being two-tailed. Blinding during data analysis was deemed unlikely due to extreme proportions.

## Results

### Sample definition and identification of RFC1 *expansion in CIAP*

**Fig. 1:**
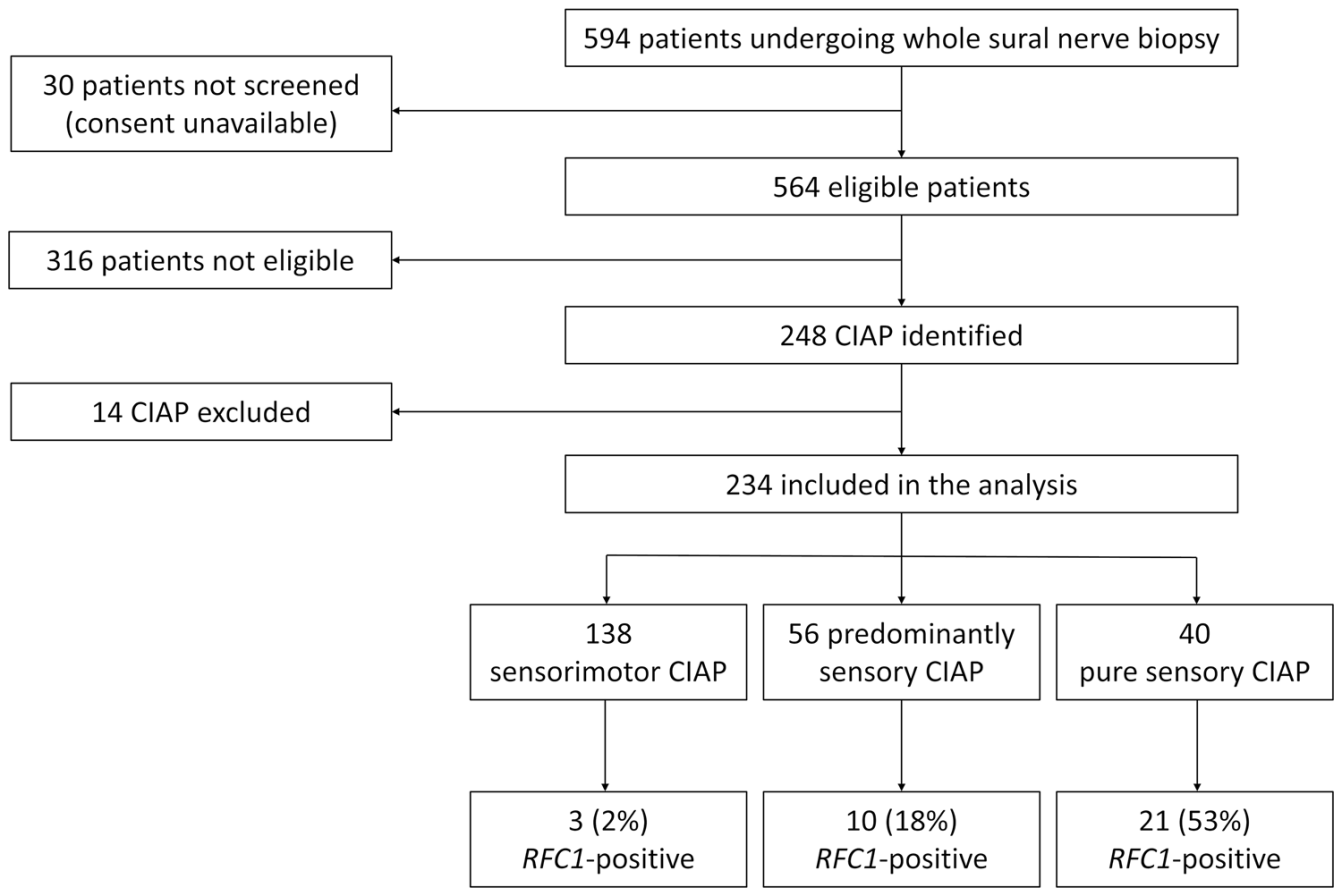
Study flow diagram

Consent to research was available in 564 out of 594 screened patients, and inclusion criteria were not met in further 316 patients (Fig. 1). Absence of a stocked DNA sample led to exclusion of 14 CIAP patients. Ultimately, 234 CIAP patients were included in the analysis. Birthplace was Northern Italy in 189, Central Italy in 17, Southern Italy in 22, other european states in 3 and extra-european in 3 (one each from Northern Africa, South America and India). Forty patients presented with pure sensory, 56 with predominantly sensory and 138 with mixed sensorimotor symptoms. The three groups were similar regarding disease duration at biopsy and sex distribution, although a non-significant lower age at onset in the pure sensory group compared to sensorimotor cases was observed (Table 1).

**Table 1:** Demographic and *RFC1* intron repeat expansion mutation status according to clinical phenotype

	Pure sensory (*n* = 40)	Predominantly sensory (*n* = 56)	Sensorimotor (*n* = 138)	*P* value	Effect size
Age at onset, median (IQR), years	58.3 (50.5–65.0)	62.5 (52.0–69.0)	64.0 (55.0–70.0)	0.05^a^	
Disease duration, median (IQR), months	36.0 (24.0–66.0)	24.0 (18.0–60.0)	48.0 (24.0–72.0)	0.23	
Females, *n* (%, CI 95%)	11 (28%, 15–44%)	23 (41%, 28–55%)	36 (26%, 19–34%)	0.11	*φ*_c_ = 0.137
AAAAG^exp^, *n* (%, CI 95%)	1 (3%, 0–13%)	3 (5%, 1–15%)	5 (4%, 1–8%)	0.80	*φ*_c_ = 0.049
AAAGG^exp^, *n* (%, CI 95%)	0 (0–9%)	0 (0–6%)	1 (1%, 0–4%)	> 0.99	*φ*_c_ = 0.055
AAGGG^exp^, *n* (%, CI 95%)	21 (53%, 36–68%)	10 (18%, 9–30%)	3 (2%, 0–6%)	**< 0.001**^**b**^	***φ***_**c**_** = 0.523**
Compound or non-canonical expansion, *n* (%, CI 95%)	1 (3%, 0–13%)	4 (7%, 2–17%)	4 (3%, 1–7%)	0.32	*φ*_c_ = 0.096

Tests reaching statistical significance are presented in bold

*IQR* interquartile range

^a^On Dunn-Bonferroni post-hoc test no pairwise comparison between groups reached the significance value

^b^On post-hoc *z*-test for independent proportions with Bonferroni correction each pairwise comparison between groups reached the significance value

**Fig. 2:**
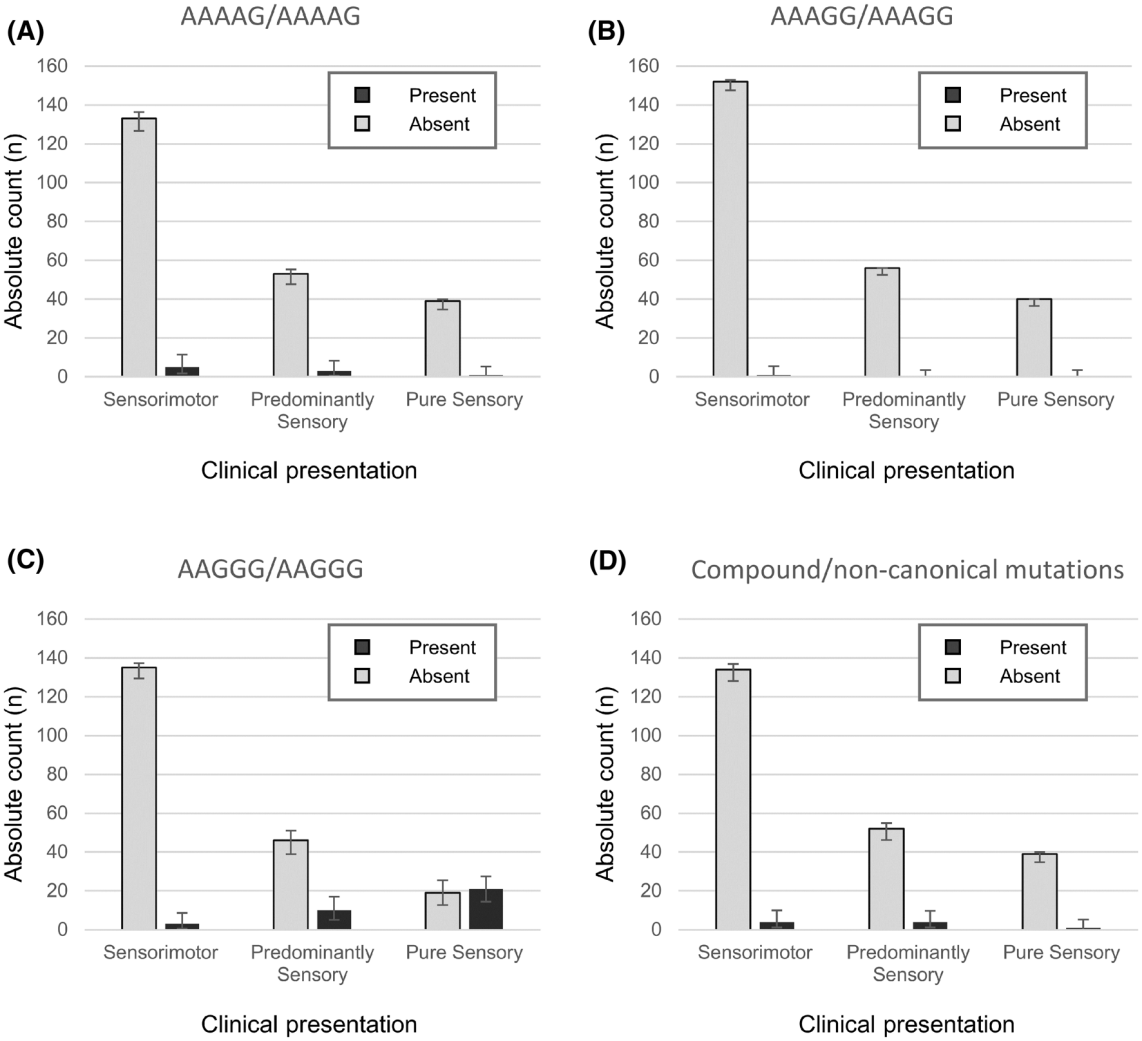
Prevalence of *RFC1* expansion mutations according to clinical presentation. Bar charts showing carriers of biallelic **a** AAAAG, **b** AAAGG, **c** AAGGG and **d** compound or non-canonical pentanucleotide *RFC1* expansion mutations in patients with Chronic Idiopathic Axonal Polyneuropathy grouped by clinical presentation. Error bar: 95% CI for within-group proportions

Clinical presentation was associated with *RFC1* expansion mutation status (*φ*_c_ = 0.381, *P* < 0.001): the *RFC1*-positive/biallelic AAGGG^exp^ occurred in 21/40 (53%, CI 95% 36–68%) pure sensory CIAP, 10/56 (18%, CI 95% 9–30%) predominantly sensory and 3/138 (2%, CI 95% 0–6%) sensorimotor neuropathy cases (Fig. 2). The same was not observed in cases with biallelic AAAAG^exp^, AAAGG^exp^ and compound or non-canonical expansions. All AAGGG^exp^ and all but three non-mutated patients were Caucasian.

### Clinical characteristics of RFC1-positive patients

Compared to non-mutated (Table 2), *RFC1*-positive patients showed a similar age at onset (median 61.0 vs 63.0 years, *R* = − 0.091, *P* = 0.17), disease duration at biopsy (median 48.0 vs 36.0 months, *R* = 0.052, *P* = 0.43) and sex distribution (female 38% vs 29%, POR 1.55 CI 95% 0.73–3.31, *P* = 0.25). Only a minority of cases appeared to be familiar (13% vs 18%, POR 0.72 CI 95% 0.23–2.23, *P* = 0.56). Consanguinity up to the third degree was not reported in any patient. *RFC1*-positive patients showed more often a positive Romberg sign (85% vs 60%, POR 3.95 CI 95% 1.47–10.6, *P* = 0.004) associated with sensory ataxia (*τ*_b_ = 0.254, *P* < 0.001) and relatively spared DTR (median total score 18.0/24 vs 11.5/24, *R* = 0.275, *P* = 0.001) compared to non-mutated, even though clinical examination did not disclose a relevant difference in tested sensory modalities (median SMSS 28.5/40 vs 30.0/40, *R* = − 0.054, *P* = 0.80). In *RFC1*-positives, a lower limb greater than upper limb involvement was evident in both sensory (median SMSS upper limbs 19.0 vs lower limbs 9.5, *R* = 0.873, *P* < 0.001) and DTR examination (median DTR score upper limbs 12.0 vs lower limbs 6.0, *R* = 0.738, *P* < 0.001).

**Table 2:** Selected demographic, clinical and instrumental data according to pathogenetic *RFC1* repeat expansion mutation status

	*RFC1*-positive (*n* = 34)	Non-mutated (*n* = 200)	*P* value	Effect size
Age at onset, median (IQR), years	61.0 (52.5–65.0)	63.0 (53.5–70.0)	0.17	*R* = − 0.091
Disease duration, median (IQR), months	48.0 (24.0–72.0)	36.0 (24.0–72.0)	0.43	*R* = 0.052
Female, *n* (%)	13 (38%)	57 (29%)	0.25	POR 1.55 (0.73–3.31)
Familiar cases, *n*/*n* (%)	4/30 (13%)	27/153 (18%)	0.56	POR 0.72 (0.23–2.23)
Same generation	3/30 (10%)	9/153 (6%)	^a^	
Multiple generations	1/30 (3%)	18/153 (12%)	^a^	
Clinical features, *n* (%)				
Paresthesias	27 (79%)	118 (59%)	**0.02**	**POR 2.68 (1.11–6.45)**
Romberg sign	29 (85%)	119 (60%)	**0.004**	**POR 3.95 (1.47–10.6)**
Cerebellar signs	3 (9%)	8 (4%)	^a^	
Nystagmus	2 (6%)	0	^a^	
Vestibulopathy	0	1 (1%)	^a^	
Autonomic disturbances	12 (35%)	15 (8%)	**< 0.001**	**POR 6.73 (2.79–16.2**)
Dysphagia	2 (6%)	8 (4%)	^a^	
Pyramidal signs	5 (15%)	20 (10%)	0.41	POR 1.55 (0.54–4.46)
Cough	3 (9%)	0	^a^	
Restless legs syndrome	2 (6%)	1 (1%)	^a^	
Pes cavus	12 (35%)	94 (47%)	0.20	POR 0.62 (0.29–1.31)
Hammer toes	5 (15%)	52 (26%)	0.16	POR 0.49 (0.18–1.33)
Pain	20 (59%)	106 (53%)	0.53	POR 1.27 (0.61–2.65)
DTR sum, median (IQR), points	18.0 (11.8–24.0)	11.5 (4.0–16.0)	**0.001**	***R = 0.275***
Ataxia score, *n* (%)				
0	5 (15%)	81 (41%)	**< 0.001**	***τ***_**b**_** = 0.254**
1	4 (12%)	60 (30%)		
2	22 (65%)	47 (24%)		
3	2 (6%)	9 (5%)		
4	1 (3%)	3 (2%)		
SMSS, median/max (IQR), points	28.5/40 (22.0–32.0)	30.0/40 (26.0–32.0)	0.80	*R* = − 0.054
Superficial sensation	10.0/16 (10.0–12.0)	12.0/16 (10.0–14.0)	0.09	*R* = − 0.123
Vibration perception	11.5/16 (7.0–14.0)	10.0/16 (8.0–12.0)	0.54	*R* = 0.037
Joint position	8.0/8 (5.0–8.0)	8.0/8 (6.0–8.0)	0.29	*R* = − 0.072
Nerve conduction studies, *n*/*n* (%)				
Axonal	34/34 (100%)^b^	135/194 (70%)^b^	**< 0.001**	***φ***_**c**_** = 0.247**
Demyelinating	0	8/194 (4%)		
Mixed	0^b^	51/194 (26%)^b^		
Sural SNAP, median (IQR), *μ*V	0 (0–0)	0 (0–3.20)	**0.003**	***R = − 0.199***
Cerebrospinal fluid, *n*/*n* (%)				
Hyperproteinorrachia	1/21 (5%)	54/113 (48%)	**< 0.001**	**POR 0.05 (0.01–0.42)**
White blood cells > 5	1/21 (5%)	0/113	^a^	

Tests reaching statistical significance are presented in bold

*SMSS* Sensory Modality Sum Score, *DTR* Deep Tendon Reflexes, *SNAP* Sensory Nerve Action Potential, *IQR* interquartile range; for categorial variables, the denominator is mentioned only in case of missing data at a group level, elsewhere assume the group total as denominator

^*a*^Not tested due to extreme counts;

^b^On Bonferroni post-hoc test *P* < 0.05 on pairwise comparison between groups

Among frequently reported features, paresthesias (79% vs 59%, POR 2.68 CI 95% 1.11–6.45, *P* = 0.02) and autonomic disturbances of sort (35% vs 8%, POR 6.73 CI 95% 2.79–16.2, *P* < 0.001) seemed to be characteristic in *RFC1*-positive patients, whereas neuropathic pain was observed in similar numbers in both groups (59% vs 53%, POR 1.27 CI 95% 0.61–2.65, *P* = 0.53). Other distinctive CANVAS features, such as cerebellar signs, vestibulopathy, chronic cough, dysphagia and restless legs syndrome were present only in a small number of cases. On the other hand, foot deformities were frequently observed in both groups (see Table 2). *RFC1*-positive consistently presented axonal neuropathy on nerve conduction studies (*φ*_c_ = 0.247, *P* < 0.001) and severely reduced sural nerve SNAP (*R* = − 0.199, *P* = 0.003). Cerebrospinal fluid analysis was usually within limits**;** contrariwise mild hyperproteinorrachia was often observed in non-mutated (5% vs 48%, POR 0.05 CI 95% 0.01–0.42, *P* < 0.001).

### Pathology findings in RFC1-positive patients

**Fig. 3:**
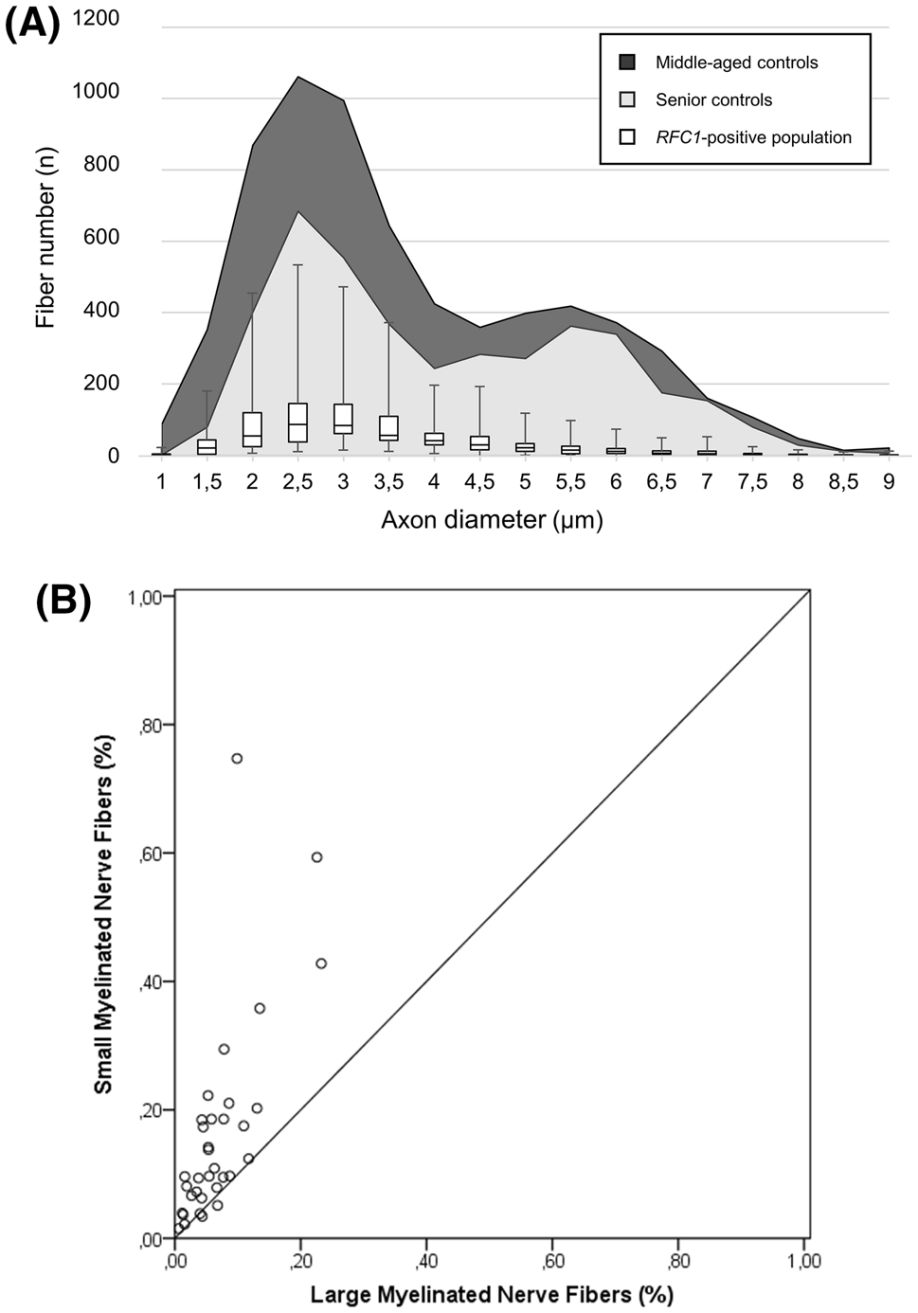
Nerve fiber distribution according to axon diameter. **a** The bimodal distribution observed in middle-aged (dark shade) and elderly controls (grey shade) is lost in *RFC1*-positive neuropathy cases (box: first and third quartile; middle bar: median; upper/lower whisker: either 1.5 interquartile range or maximum/minimum observed values); **b** scatterplot of estimated surviving large and small myelinated fibers: extreme loss of large fibers but variable involvement of small myelinated fibers is observed

Sural nerve morphometry revealed a severe loss of MF (median total surviving fibers absolute count 678.9 IQR 412.9–918.8; proportion of surviving fiber relative to controls 12.8% IQR 7.8–17.3%) with a tendency for relevant although milder involvement of small MF compared to large MF (median relative surviving small MF 10.3% IQR 6.6–18.6%; large MF 5.4% IQR 3.8–8.6%) (Fig. 3). Accordingly, pathology records described an involvement of large, small MF and unmyelinated nerve fibers, respectively, in 100%, 97% and 85% of *RFC1*-positive patients, and 99%, 81% and 41% in other neuropathies (Table 3). Parcel involvement with substantial sparing of small MF and unmyelinated fibers was thus more frequent in non-mutated patients (group difference in the involvement of unmyelinated fibers POR 8.52 CI 95% 3.17–22.9, *P* < 0.001; small MF POR 7.74 CI 95% 1.03–58.4, *P* = 0.02; large MF: not tested due to extreme values). Nerve morphometry did not differ between patients presenting with pain, paresthesias or motor symptoms.

**Table 3:** Neuropathological features according to pathogenetic *RFC1* repeat expansion mutation status

	*RFC1*-positive (*n* = 34)	Non-mutated (*n* = 200)	*P* value	Effect size
Involvement of selected nerve fibers, *n* (%)				
Large myelinated nerve fibers	34 (100%)	198 (99%)	^a^	
Small myelinated nerve fibers	33 (97%)	162 (81%)	**0.02**	**POR 7.74 (1.03–58.4)**
Unmyelinated nerve fibers	29 (85%)	81 (41%)	**< 0.001**	**POR 8.52 (3.17–22.9)**
Active degeneration, *n* (%)				
Absent	16 (47%)	83 (42%)	0.47	*τ*_b_ = − 0.046
Rare	17 (50%)	106 (53%)		
Occasional	1 (3%)	9 (5%)		
Diffuse	0	2 (1%)		
Regeneration clusters, *n* (%)				
Absent	17 (50%)	20 (10%)	**< 0.001**	***τ***_**b**_** = − 0.362**
Rare	16 (47%)	92 (46%)		
Occasional	1 (3%)	64 (32%)		
Diffuse	0	24 (12%)		
Microangiopathy, *n* (%)				
Absent	17 (50%)	117 (59%)	0.34	*τ*_b_ = − 0.060
Occasional	11 (32%)	54 (27%)		
Diffuse	6 (18%)	28 (14%)		

Tests reaching statistical significance are presented in bold

^a^Not tested due to extreme counts

**Fig. 4:**
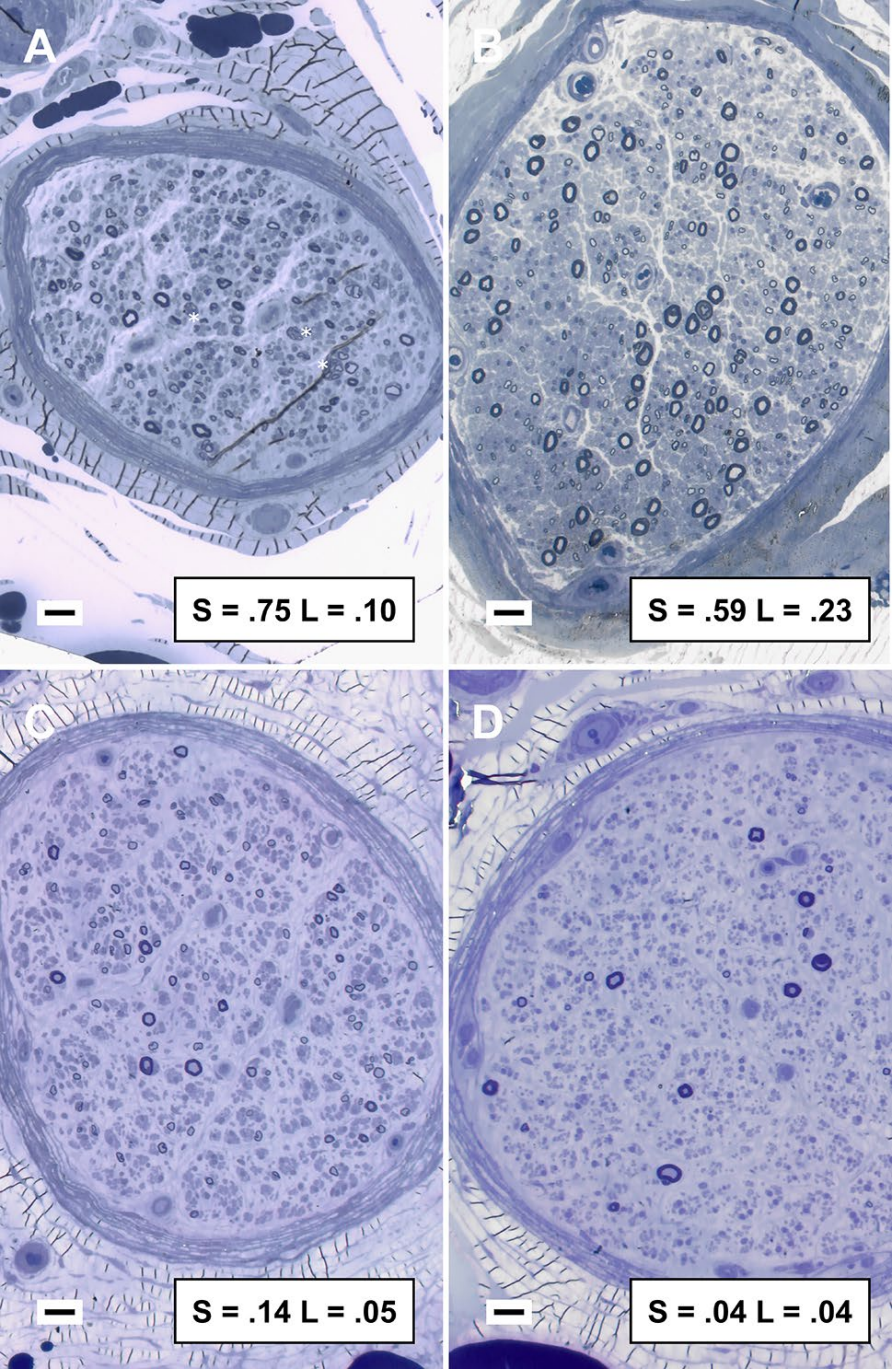
Representative cases from sural nerve pathology studies. Sural nerve biopsy revealed a diffuse depletion of large myelinated fibers that was similar in severity between patients, instead small myelinated fibers appeared relatively preserved in less affected nerves (**a**–**b**). Regeneration clusters were observed in a single instance and associated with mild disease (asterisks, **a**). Active degeneration was uncommon. Toluidine blue-stained semithin cross sections of sural nerve, bar = 20 µm; inbox: fraction of surviving small (S) and large (L) myelinated fibers compared to control average

Surviving total, small and large MF correlated with age at biopsy (*ρ* = − 0.557 *P* = 0.001, *ρ* = − 0.534 *P* = 0.001, *ρ* = − 0.634 *P* < 0.001 for total, small and large MF), amended SMSS (*ρ* = 0.366 *P* = 0.03, *ρ* = 0.338 *P* = 0.05, *ρ* = 0.301 *P* = 0.08) and ataxia severity (*ρ* = − 0.430 *P* = 0.01, *ρ* = − 0.465 *P* = 0.006, *ρ* = − 0.357 *P* = 0.04), but not DTR score. Both active degeneration (median 0.9% IQR 0.2–1.4%) and regeneration clusters (1.0% IQR 0–2.1%) were an uncommon finding, the latter being less represented than in non-mutated neuropathies (*τ*_b_ = − 0.362, *P* < 0.001). In a single instance, a 68-years-old male with a 36-month history of progressive frank sensory ataxia and moderate sensory impairment (SMSS 32) and past, non-active history of alcohol abuse, we observed frank regenerative changes associated with moderate axonal loss (estimated total MF 2941, surviving large MF 9.9%, small MF 74.7%) (Fig. 4a).

## Discussion

The distinctive biallelic *RFC1* intronic mutation and expansion of the pentanucleotide AAGGG associated with CANVAS is a striking finding in this study investigating a cohort of selected CIAP patients. The mutation was identified in 15% of the selected patients’ population; it occurred in the majority (53%) of patients with pure sensory forms and was not uncommon (18%) even in cases with minor motor involvement. The clinical picture of a slowly progressive sensory ataxia associated with a length-dependent neuropathy with hypoesthesia, neuropathic pain and autonomic disturbances observed in our *RFC1*-positive population shares remarkable similarity to the peripheral involvement described in previous CANVAS series [8, 14], although frequently reported also in other neuropathies. Paradoxical retention of DTR in face of relevant sensory impairment, on the other hand, is a peculiar occurrence observed in our series as well as in other CANVAS cases [8]. Muscle spindle afferents are responsible for DTR and play a major role in proprioception, in concert with cutaneous and Golgi tendon organ afferents [15, 16]. A dissociated involvement of sensory afferents with greater impairment of cutaneous compared to muscle spindle innervation has been previously described [17, 18], and possibly underlies the paradoxical DTR retention in spite of frank rombergism. A similar dissociate impairment of sensory modalities has been proved also for vestibular responses [19].

Cerebellar and vestibular symptoms, the remaining features of the variegate CANVAS clinical phenotype, were otherwise absent at pre-biopsy visit, even though a considerable lapse of time had passed since onset.

In the largest retrospective cohort of *RFC1*-mutated CANVAS patients described so far, features of sensory neuro(no)pathy were accordantly observed in most patients and often reported since onset, while the involvement of other systems manifested later in the history of the disease or emerged only from extensive instrumental investigations [8]. An archetypic pattern of progression could then explain the lower yield of genetic investigations for *RFC1* mutation expansion observed in previous reports on patients with adult-onset ataxia of unknown etiology [20–26] or Multiple System Atrophy [20, 23] compared to ours, even though the differences in study design prevent valid comparisons.

On pathology, we observed a marked and diffuse loss of large MF in all patients and, to a milder degree, of small MF. On the contrary, unmyelinated nerve fibers involvement was less frequently reported on our qualitative ultrastructural studies. Similar findings were reported on qualitative [14] and morphometry analysis [8] in smaller case series. Greater involvement of large MF suits both the clinical presentation as an ataxic neuropathy and the decrease in SNAP amplitude observed even in paucisymptomatic patients. Notably, the normal sural nerve rarely harbors muscle spindle afferents as it commonly lacks muscular branches, so that its biopsy does not provide information on group Ia sensory fibers. Demonstration of survival of these fibers relies on clinical and neurophysiological studies in life, while necropsy examinations will be required for pathological confirmation.

Absence of relevant signs of axonal regeneration point**s** to a neuronopathy as the more probable mechanism of degeneration, in agreement with two histopathological case series reporting neuronal loss in dorsal root ganglia [27] and in vestibular, geniculate and trigeminal ganglia [28], although at odd with the length-dependent characteristic observed.

Based on these marked similarities, we confidently conclude that we identified either an early manifestation of full CANVAS or, less likely, a *forme-fruste* variant. Follow-up is needed to address the development of the full-blown syndrome along the disease course.

Our findings suggest that, even in the absence of the classic CANVAS triad, several elements should alert the clinician in middle-aged patients with a history of a slowly progressive sensory neuro(no)pathy, in particular: (a) sensory axonal neuropathy on NCS; (b) paradoxical retention of DTR in face of relevant sensory impairment; (c) dramatic involvement of both myelinated and unmyelinated nerve fibers on sural nerve biopsy. On the other hand, lack of a definite family history, the presence of minor motor deficits or foot deformities should not avert from investigating a patient for the *RFC1* expansion**s**.

### Strengths and limitations

In our setting, heterogeneity in clinical assessment and pathology evaluation of a large cohort of patients has been minimized by means of operator consistency and shared practices. Neurophysiological evaluation, on the other hand, had been assessed by multiple neurophysiologists in different clinics and thus we cannot rule out inconsistencies on NCS conclusions. Although extensive, in this real-world scenario routine neurological examination was still aimed at the referral question and could have led to underrepresentation of ancillary clinical features. Advanced bedside clinical maneuvers to test the vestibular system were not customary and, similarly, assessment of autonomic disturbances was often limited to an evaluation by interview, leading to the identification of patients with remarkable symptoms only. Moreover, inquiry for chronic cough became relevant just recently after being described as a defining symptom in CANVAS. The authors acknowledge that implementation of further testing could possibly lead the wary clinician to identify preclinical involvement in part of these patients. Regarding our study, blinding during data extraction still secured an unbiased distribution of these characteristics between *RFC1*-positive and non-mutated participants.

The addition of pathological criteria to routine examinations in selecting CIAP patients may represent a double-edged sword: on the one hand, it may have ruled out other confounding acquired or genetic causes of neuropathy; on the other, it may have introduced a bias in the selection of the study population. A sural nerve biopsy is more likely offered to patients with a suspected treatable cause, absence of an overt family history, younger onset, more severe or rapidly progressive course; however, the relevance of this potential bias is probably limited because those features do not characterize the *RFC1*-positive patients depicted by previous works [8].

Clinical and/or neurophysiological involvement of the sural nerve is required for the bioptic procedure and could have biased the phenotypical characterization due to an enrichment in patients with distal involvement. However, we note that previous works on full CANVAS also showed a similar presence of length-dependent neuro(no)pathy [14].

## Conclusions

The biallelic *RFC1* AAGGG repeat expansion is a frequent cause of adult-onset, slowly progressive, pure or prevalently sensory axonal neuro(no)pathies of unknown cause. Future natural history studies will clarify whether the chronic axonal neuro(no)pathy necessarily heralds the development of a full-blown CANVAS or if it may remain for a long time as the dominant phenotype associated to the *RFC1* AAGGG repeat expansion**.**

## Supplementary Information

Below is the link to the electronic supplementary material.

Supplementary Online Resource 1. Schematic representation of RFC1 intron 2 (DOCX 36 KB)

Supplementary Online Resource 2. Flanking standard PCR (DOCX 55 KB)

Supplementary Online Resource 3. Primer sequences used in RFC1 gene analysis (DOCX 19 KB)

Supplementary Online Resource 4. Repeat primed PCR (DOCX 180 KB)

Supplementary Online Resource 5. Genes included in the Next Generation Sequencing panel (DOCX 20 KB)

## Data Availability

Anonymized data will be shared on request from qualified investigators.
