# Supplementary material for: RFC1 AAGGG repeat expansion masquerading as Chronic Idiopathic Axonal Polyneuropathy
Source: J Neurol. 2021 Apr 21;268(11):4280–90. doi: 10.1007/s00415-021-10552-3 (PMC8505379; doi:10.1007/s00415-021-10552-3)
Supplement: Supplementary file 1 — Supplementary Online Resource 1. Schematic representation of RFC1 intron 2 (DOCX 36 KB) [file 415_2021_10552_MOESM1_ESM.docx]

***RFC1* AAGGG repeat expansion masquerading as Chronic Idiopathic Axonal Polyneuropathy**

# **Journal of Neurology**

Matteo Tagliapietra M.D.^1^ (0000-0002-3048-1453), Davide Cardellini M.D.^1^, Moreno Ferrarini Ph.D.^1^ (0000-0001-8768-7922), Silvia Testi Ph.D.^1^ (0000-0003-0267-0000), Sergio Ferrari M.D.^1^ (0000-0003-3855-5135), Salvatore Monaco M.D.^1^ (0000-0003-3191-8597), Tiziana Cavallaro M.D.^1^ (0000-0002-7851-6408) and Gian Maria Fabrizi M.D. Ph.D.^1^ (0000-0001-6804-0226)

^1^ Department of Neurosciences, Biomedicine, and Movement Sciences, University of Verona, Piazzale L.A. Scuro, 10, 10, 37134, Verona, VR, Italy

Corresponding author: Prof. Gian Maria Fabrizi, Policlinico G.B. Rossi, P.le L.A. Scuro 10, 37134 Verona, Italy

Telephone: +39 045 8124286, Fax: +39 0458027492, E-mail: [gianmaria.fabrizi@univr.it](mailto:gianmaria.fabrizi@univr.it)

Wild type

***RFC1***

Exon 3

Exon 2

(AAAAG)_11_

(AAAAG)_exp_ or (AAAGG)_exp_

not pathogenic (AAAAG) or (AAAGG) expansion

(AAGGG)_exp_

CANVAS-associated (AAGGG) expansion

**Online Resource 1. Schematic representation of *RFC1* intron 2.**

Blue rectangles refer to *RFC1* exons 2 and 3; blue arrows show gene orientation; the red rectangle refers to intron 2 region which may undergo pathogenic or not pathogenic expansions; the green rectangle refers to the Alu element region (AluSx3); black arrows show the position of primers used for flanking standard PCR. Dot black lines delimit the amplified region.
