# Supplementary material for: RFC1 AAGGG repeat expansion masquerading as Chronic Idiopathic Axonal Polyneuropathy
Source: J Neurol. 2021 Apr 21;268(11):4280–90. doi: 10.1007/s00415-021-10552-3 (PMC8505379; doi:10.1007/s00415-021-10552-3)
Supplement: Supplementary file 3 — Supplementary Online Resource 3. Primer sequences used in RFC1 gene analysis (DOCX 19 KB) [file 415_2021_10552_MOESM3_ESM.docx]

***RFC1* AAGGG repeat expansion masquerading as Chronic Idiopathic Axonal Polyneuropathy**

# **Journal of Neurology**

Matteo Tagliapietra M.D.^1^ (0000-0002-3048-1453), Davide Cardellini M.D.^1^, Moreno Ferrarini Ph.D.^1^ (0000-0001-8768-7922), Silvia Testi Ph.D.^1^ (0000-0003-0267-0000), Sergio Ferrari M.D.^1^ (0000-0003-3855-5135), Salvatore Monaco M.D.^1^ (0000-0003-3191-8597), Tiziana Cavallaro M.D.^1^ (0000-0002-7851-6408) and Gian Maria Fabrizi M.D. Ph.D.^1^ (0000-0001-6804-0226)

^1^ Department of Neurosciences, Biomedicine, and Movement Sciences, University of Verona, Piazzale L.A. Scuro, 10, 10, 37134, Verona, VR, Italy

Corresponding author: Prof. Gian Maria Fabrizi, Policlinico G.B. Rossi, P.le L.A. Scuro 10, 37134 Verona, Italy

Telephone: +39 045 8124286, Fax: +39 0458027492, E-mail: [gianmaria.fabrizi@univr.it](mailto:gianmaria.fabrizi@univr.it)

| **Online resource 3. Primer sequences used in *RFC1* gene analysis.** | | |
| --- | --- | --- |
| Primer name | Position (hg19) | Sequence |
| RFC1_FW | 39350151-39350123 | 5’-tcaagtgatactccagctacaccgttgc |
| RFC1_RV | 39349804-39349829 | 5’-gtgggagacaggccaatcacttcag |
| RFC1_(AAAAG)_RV | undefined | 5’-CAGGAAACAGCTATGACCAACAGAGCAAGACTCTGTTTCAAAAAAGAAAAGAAAAGAAAAGAAAA |
|  |  | 5’-CAGGAAACAGCTATGACCAACAGAGCAAGACTCTGTTTCAAAAAGAAAAGAAAAGAAAAGAAAA |
|  |  | 5’-CAGGAAACAGCTATGACCAACAGAGCAAGACTCTGTTTCAAAAGAAAAGAAAAGAAAAGAAAA |
| RFC1_(AAAGG)_RV | undefined | 5’-CAGGAAACAGCTATGACCAACAGAGCAAGACTCTGTTTCAAAAAAGGAAAGGAAAGGAAAGGAAA |
|  |  | 5’-CAGGAAACAGCTATGACCAACAGAGCAAGACTCTGTTTCAAAAAGGAAAGGAAAGGAAAGGAAA |
|  |  | 5’-CAGGAAACAGCTATGACCAACAGAGCAAGACTCTGTTTCAAAAGGAAAGGAAAGGAAAGGAAA |
| RFC1_(AAGGG)_RV | undefined | 5’-CAGGAAACAGCTATGACCAACAGAGCAAGACTCTGTTTCAAAAAAGGGAAGGGAAGGGAAGGGAA |
|  |  | 5’-CAGGAAACAGCTATGACCAACAGAGCAAGACTCTGTTTCAAAAAGGGAAGGGAAGGGAAGGGAA |
|  |  | 5’-CAGGAAACAGCTATGACCAACAGAGCAAGACTCTGTTTCAAAAGGGAAGGGAAGGGAAGGGAA |
| Anchor | M13 RV | 5’-CAGGAAACAGCTATGACC |

Primer sequences used for the analysis of the *RFC1* gene (reference sequence NM_002913.5) according to Cortese et al.^2^ Flanking standard PCR was done using the RFC1_Fw and RFC1_RV primers. Repeat-primed PCR was done using different combinations of primers: fluorescently labelled RFC1-FW plus the anchor were differentially combined with RFC1_(AAAAG)_RV, RFC1_(AAAGG)_RV and RFC1_(AAGGG)_RV which recognize respectively the non-pathogenic (AAAAG) expansion, the non-pathogenic (AAAGG), and the CANVAS-associated (AAGGG) expansion.
