# Supplementary material for: RFC1 AAGGG repeat expansion masquerading as Chronic Idiopathic Axonal Polyneuropathy
Source: J Neurol. 2021 Apr 21;268(11):4280–90. doi: 10.1007/s00415-021-10552-3 (PMC8505379; doi:10.1007/s00415-021-10552-3)
Supplement: Supplementary file 5 — Supplementary Online Resource 5. Genes included in the Next Generation Sequencing panel (DOCX 20 KB) [file 415_2021_10552_MOESM5_ESM.docx]

***RFC1* AAGGG repeat expansion masquerading as Chronic Idiopathic Axonal Polyneuropathy**

# **Journal of Neurology**

Matteo Tagliapietra M.D.^1^ (0000-0002-3048-1453), Davide Cardellini M.D.^1^, Moreno Ferrarini Ph.D.^1^ (0000-0001-8768-7922), Silvia Testi Ph.D.^1^ (0000-0003-0267-0000), Sergio Ferrari M.D.^1^ (0000-0003-3855-5135), Salvatore Monaco M.D.^1^ (0000-0003-3191-8597), Tiziana Cavallaro M.D.^1^ (0000-0002-7851-6408) and Gian Maria Fabrizi M.D. Ph.D.^1^ (0000-0001-6804-0226)

^1^ Department of Neurosciences, Biomedicine, and Movement Sciences, University of Verona, Piazzale L.A. Scuro, 10, 10, 37134, Verona, VR, Italy

Corresponding author: Prof. Gian Maria Fabrizi, Policlinico G.B. Rossi, P.le L.A. Scuro 10, 37134 Verona, Italy

Telephone: +39 045 8124286, Fax: +39 0458027492, E-mail: [gianmaria.fabrizi@univr.it](mailto:gianmaria.fabrizi@univr.it)

| **Online Resource 5. Genes included in the Next Generation Sequencing panel.** | | | | |
| --- | --- | --- | --- | --- |
|  | Gene | Reference sequence | Size (bp) | Design covered (%) |
| 1 | APOA1 | NM_000039.3 | 864 | 100 |
| 2 | ATP1A1 | NM_000701.8 | 3324 | 100 |
| 3 | BSCL2 | NM_001122955 | 1609 | 100 |
| 4 | DNM2 | NM_001005361.3 | 3192 | 100 |
| 5 | EGR2 | NM_000399.5 | 1471 | 100 |
| 6 | GARS1 | NM_002047.4 | 2560 | 100 |
| 7 | GDAP1 | NM_018972.4 | 1197 | 100 |
| 8 | GJB1+5’-UTR | NM_000166.6 | 1697 | 100 |
| 9 | GSN | NM_198252.3 | 2949 | 100 |
| 10 | HINT1 | NM_005340.7 | 441 | 100 |
| 11 | HSPB1 | NM_001540.5 | 678 | 100 |
| 12 | HSPB8 | NM_014365.3 | 651 | 100 |
| 13 | LITAF | NM_001136472.1 | 648 | 100 |
| 14 | LRSAM1 | NM_138361 | 2652 | 99.8 |
| 15 | MFN2 | NM_014874.4 | 2614 | 100 |
| 16 | MME | NM_007289.4 | 2693 | 100 |
| 17 | MPZ | NM_000530.8 | 1059 | 100 |
| 18 | NEFH | NM_021076.4 | 3143 | 100 |
| 19 | NEFL | NM_006158.5 | 1713 | 100 |
| 20 | PMP2 | NM_002677.5 | 439 | 100 |
| 21 | PMP22 | NM_000304.4 | 563 | 100 |
| 22 | RAB7A | NM_004637.6 | 724 | 100 |
| 23 | SH3TC2 | NM_024577.4 | 4207 | 100 |
| 24 | TTR | NM_000371.4 | 524 | 100 |

Gene panel used for mutational screening by Ion PGM Dx System. Mean depth coverage 30X. Experimental coverage: 100% for all genes but NEFH (coverage = 88%).
